# The Influence of Electrolytic Concentration on the Electrochemical Deposition of Calcium Phosphate Coating on a Direct Laser Metal Forming Surface

**DOI:** 10.1155/2017/8610858

**Published:** 2017-01-29

**Authors:** Qianyue Sun, Yuhui Yang, Wenjing Luo, Jinghui Zhao, Yanmin Zhou

**Affiliations:** ^1^Department of Dental Implantology, School and Hospital of Stomatology, Jilin University, Changchun, Jilin Province 130021, China; ^2^Key Laboratory of Oral Medicine, Guangzhou Institute of Oral Disease, Stomatology Hospital of Guangzhou Medical University, Guangzhou 510140, China; ^3^Department of Orthopaedics, China-Japan Union Hospital of Jilin University, Changchun, Jilin Province 130033, China

## Abstract

A calcium phosphate (CaP) coating on titanium surface enhances its biocompatibility, thus facilitating osteoconduction and osteoinduction with the inorganic phase of the human bone. Electrochemical deposition has been suggested as an effective means of fabricating CaP coatings on porous surface. The purpose of this study was to develop CaP coatings on a direct laser metal forming implant using electrochemical deposition and to investigate the effect of electrolytic concentration on the coating's morphology and structure by X-ray diffraction, scanning electron microscopy, water contact angle analysis, and Fourier transform infrared spectroscopy. In group 10^−2^, coatings were rich in dicalcium phosphate, characterized to be thick, layered, and disordered plates. In contrast, in groups 10^−3^ and 10^−4^, the relatively thin and well-ordered coatings predominantly consisted of granular hydroxyapatite. Further, the hydrophilicity and cell affinity were improved as electrolytic concentration increased. In particular, the cells cultured in group 10^−3^ appeared to have spindle morphology with thick pseudopodia on CaP coatings; these spindles and pseudopodia strongly adhered to the rough and porous surface. By analyzing and evaluating the surface properties, we provided further knowledge on the electrolytic concentration effect, which will be critical for improving CaP coated Ti implants in the future.

## 1. Introduction

Many dental and orthopedic implants are manufactured from titanium (Ti) as it has excellent mechanical properties, such as corrosion resistance and biocompatibility [1]. However, conventional Ti implants also have some limitations. Mismatched elastic modulus between the implant and native bone leads to stress shielding and implant failure [2]. Since pure Ti implant contains an inert metal and lacks osteoinduction, it mechanically interlocks with the bone surface without forming a chemical bond [3]. Therefore, many researchers have attempted to develop surface treatment methods that improve the efficacy and bone-bonding ability of Ti dental implants.

Rapid prototyping (RP) technologies can directly fabricate individualized products with defined morphology and structure on the basis of virtual three-dimensional (3D) data. According to a computer assisted design (CAD) file, direct laser metal forming (DLMF) has been used to produce laser-sintered titanium implants, which are usually known as TixOs® implants (Leader-Novaxa, Milan, Italy) [4–6]. The surface of this implant is characterized by a 3D network of interconnected pores that can significantly facilitate cell growth and bone deposition. The surface morphology and elastic modulus of these implants are also designed to resemble the structure and morphology of porous alveolar bone [7]. Moreover, the core of such implants retains the excellent mechanical properties of Ti. Hence, these implants have high overall strength and rigidity.

Many research studies [8] have given promising results showing that depositing calcium phosphate (CaP) coating on the surface of Ti implants can improve the bioinert surface of titanium alloys. Moreover, the coating's structural and chemical properties are similar to those of human bone tissue [9–12]. CaP coating, as a main compound in the inorganic bone matrix, significantly improves the biocompatibility, osteoconduction, and osteoinduction of Ti implants, as shown in the study by de Jonge et al. [13]. Furthermore, the release of chemical elements (Ca and P) from the coating can be cleared through a series of metabolic pathways [14]. After fixing the Ti implant, the CaP coating can form chemical bonds with adjacent bone tissue [15], thus forming a scaffold to facilitate the formation of new bone [16]. The CaP coating is remodeled into new bone as it eventually undergoes degradation; this new bone absorbs the less stable CaP phases of the coating. In addition, many researchers have claimed that the highly crystalline form of pure hydroxyapatite (HAP) is an effective bioactive coating on Ti surfaces [17].

Different methods have been developed for coating CaP layers onto implant surfaces, such as plasma spray [18], sol–gel [19], biomimetic [20], chemical vapor deposition [21], ion implantation [22], and electrochemical deposition [23]. Electrochemical deposition, a liquid-based process, has been suggested as an effective means of fabricating CaP coatings on intricate geometry substrates [24]. By using a lower working temperature, changes in the chemical composition and crystal structure of the substrate (normally caused by conventional sintering processes) can be avoided [23]. Meanwhile, the chemical composition, physical phases, and microstructure of substrates subjected to electrochemical deposition are controlled by parameters associated with the process of deposition, such as deposition temperature, voltage, current density, and electrolytic concentration [25]. Even electrochemical deposition was demonstrated to promptly functionalize 3D porous Ti structures with CaP layers and offer higher controllability and reproducibility on the surface coating [24, 26]. Very few studies have reported the effect of electrolytic concentration on the structure and morphology of CaP coatings.

In this study, we determined how different electrolytic concentrations affected the structure and morphology of CaP coatings on porous Ti implant surfaces. We also determined the predominant composition and cellular adhesion of CaP coatings produced by electrochemical deposition on the surface of DLMF implants.

## 2. Materials and Methods

### 2.1. Preparation of Specimens

In this experimental study, cylindrical TixOs plates (2 mm thick and 10 mm in diameter) were obtained from Leader Italia Srl (Cinisello Balsamo, Italy). Using a DLMF technique, the plates were manufactured from an alloy powder (Ti-6Al-4V), with a particle size of 25–45 *μ*m as the base material.

### 2.2. Electrochemical Deposition of CaP Coating

#### 2.2.1. Experimental Groups

Based on the concentration of Ca(NO_3_)_2_ in the aqueous solution, we classified different electrolytic concentrations into three concentration-level groups: group 10^−2^, group 10^−3^, and group 10^−4^ (Table 1). The ratio of Ca/P in electrolyte was kept constant at 1.67 to ensure the stoichiometric levels were consistent with HAP, as reported previously [27, 28].

#### 2.2.2. Experimental Procedure

In this experiment, electrochemical deposition was used to form CaP coatings on the surface of Ti plates. We initially prepared three solutions of different electrolytic concentrations. Depending on the different concentrations of (NH_4_)H_2_PO_4_ and Ca(NO_3_)_2_·4H_2_O in distilled water, we defined three experimental groups: group 10^−2^, group 10^−3^, and group 10^−4^. The bare Ti implants were used as the control group. We dissolved 0.1 mol of NaNO_3_ in the electrolytic solutions of groups 10^−3^ and 10^−4^ to increase their ionic conductivity. The acidity of the electrolytic solutions was maintained at pH 6 using ammonia water. The electrochemical deposition was conducted for 40 minutes at a constant potential of −2.5 V. During this process, the temperature was maintained at 70°C. The plate specimens and a platinum electrode were used as the cathode and anode, respectively. The distance between the electrodes was maintained at 10 mm. The potentials were recorded during the coating process. Finally, the deposits were gently rinsed with distilled water and dried overnight at room temperature.

### 2.3. Microstructure and Structural Characterization

The microstructure of deposits was analyzed by scanning electron microscopy (SEM) (XL30ESEM-FEG; FEL, Amstelveen, Netherlands). The crystal phase and constituents of calcium phosphate coating were investigated by X-ray diffraction (XRD) (D8 Advance; Bruker, Karlsruhe, Germany) and Fourier transform infrared spectroscopy (FTIR) (D8 Advance; Bruker, Karlsruhe, Germany), respectively. Further, the hydrophilic property of implants was characterized by measuring the static water contact angles at room temperature.

### 2.4. Cell Culture Experiment

According to the experimental design, the human osteoblast-like MG-63 cell line was cultured in Dulbecco's Modified Eagle Medium (DMEM), which was supplemented with 10% fetal calf serum and 1% penicillin. This cell culture was incubated at 37°C in a humidified atmosphere containing 5% CO_2_. The cells were seeded and cultured on the surface of specimens obtained from the three different groups for a time-period of 1, 3, and 7 days; the cell density was maintained at 5 × 10^4^ cells/cm^2^. After gently rinsing with phosphate-buffered saline (PBS), we fixed these specimens for 2 hours in 2.5% glutaraldehyde at 4°C. After fixing these specimens, we dehydrated them using a series of ethanol solutions of increasing concentrations: 30, 50, 70, 80, 90, 95, and 100%. These ethanol solutions were subsequently sprayed on the specimens. Finally, a SEM was used to analyze the morphology of cells cultured on these specimens.

## 3. Results

### 3.1. Morphological Analysis

**Figure 1:**
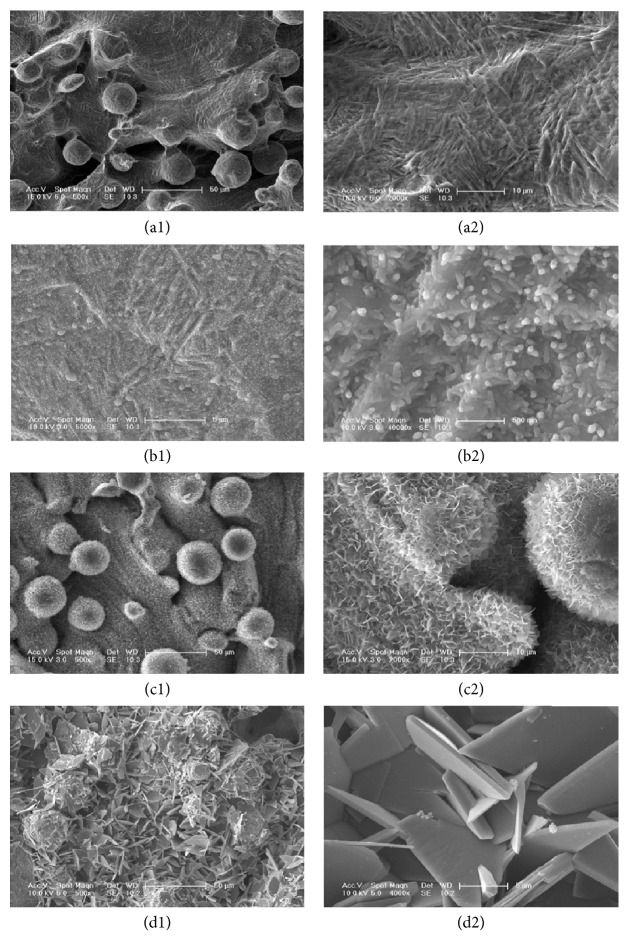
SEM micrographs of the bare Ti implant (a1) and of the CaP coating obtained using different electrolytic concentrations: group 10^−4^ (b1); group 10^−3^ (c1); and group 10^−2^ (d1). The photographs (a2–d2) were the corresponding magnified images.

The DLMF implant surface (Figure 1(a1)) was covered with spherical particles with a diameter of 30 to 50 *μ*m. The titanium spherical particles had a porous structure; the diameter of these pores varied between 200 and 300 *μ*m (Figure 1(a2)). In group 10^−4^ (Figure 1(b1)), the thin CaP coating had rod-like morphology; these tiny rods were 200 nm long and their diameters were 60–80 nm. These tiny rods randomly covered the thin surface of the CaP coating. Under large magnification (Figure 1(b2)), we found that the surface of the rods was smooth. The coating exhibited layers of crystal growth, in which larger tabular crystals were found to be superposed on thin layers of crystals. This indicated that the coating was formed by a process involving classical nucleation and growth. The surface of the specimen of group 10^−3^ (Figure 1(c1)) had a porous, well-ordered lattice, which was covered by structural laminas of CaP coatings, having cauliflower morphology. In a highly magnified image (Figure 1(c2)), we found that the coating was composed of crystal structures, having a thickness of 30–50 nm and a length of 1-2 *μ*m. The specimen of group 10^−2^ (Figure 1(d1)) had a very thick coating that was composed of disorderly deposited plates, in which the micropores were completely covered with CaP deposits. The highly magnified micrograph (Figure 1(d2)) showed that thin CaP crystals appeared as flat interwoven plates. The thickness and length of these crystals were 600 nm and 10 *μ*m, respectively.

### 3.2. Deposition Current Density

**Figure 2:**
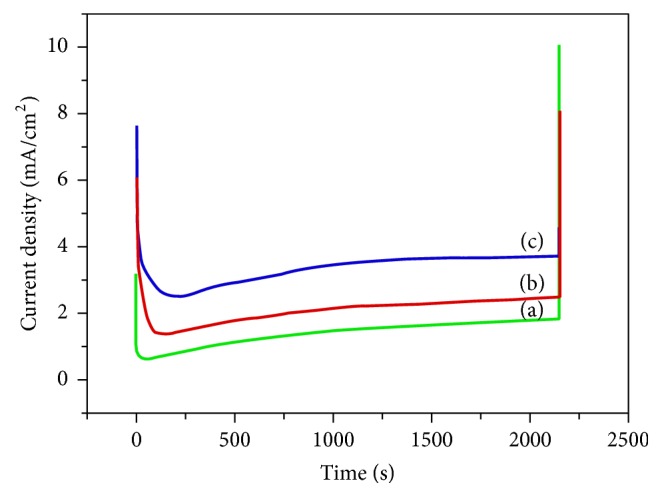
Current density during the electrochemical deposition of CaP coating using different electrolytic concentrations: group 10^−4^ (a), group 10^−3^ (b), and group 10^−2^ (c).

A potentiostatic method was used for carrying out the deposition of CaP coating.  Figure 2 illustrates the plot of electric current against the applied potential. The deposition of CaP was carried out at a constant potential. As the concentration of the electrolyte increased at the electrode surface, the electric current also showed an upward trend. A higher electric current accelerated the deposition of the electrolyte at the cathode. Although the rate of deposition improved, a higher electric current also generated excessive OH^−^ ions [29], leading to the formation of a flocculent precipitate as CaP was deposited away from the cathode. The CaP coating generated under these conditions appeared to be loose and disordered, thus causing lower adhesion strength between the coating and the surface.

### 3.3. Composition and Structural Analysis

#### 3.3.1. X-Ray Diffraction (XRD) Analysis

**Figure 3:**
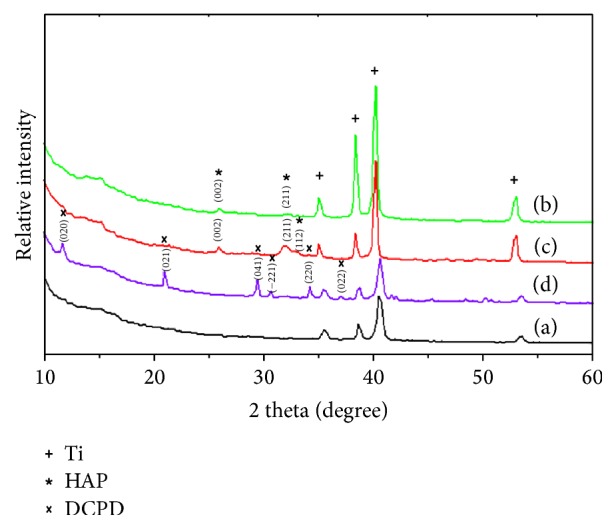
XRD pattern of the bare Ti implant specimen (a) and of the specimens coated with CaP at different electrolytic concentrations: group 10^−4^ (b); group 10^−3^ (c); and group 10^−2^ (d).

Figure 3 shows the XRD patterns of various specimens. Most clusters have components located in the 2*θ* region between 33° and 41°, which illustrated titanium oxides were presenting in the titanium interlayer [30]. After comparing these XRD patterns with the standard XRD curve of HAP (JCPDS 09-0432), we proposed that the diffraction peaks of groups 10^−3^ and 10^−4^ represented the HAP phase. The diffraction patterns of groups 10^−3^ and 10^−4^ showed peaks with the following 2*θ* values: 25.95° (002), 31.96° (211), and 49.52° (213). Therefore, we inferred from the graph that the coatings of these two groups were predominantly composed of HAP. Moreover, in groups 10^−3^ and 10^−4^, the thickness of the coating and the intensity of diffraction peaks increased when we increased the electrolytic concentration. In group 10^−2^, the electrolytic concentration predominantly produced a highly crystalline phase of dibasic calcium phosphate dihydrate (DCPD; CaHPO_4_·2H_2_O).

#### 3.3.2. Fourier Transform Infrared (FITR) Analysis

In the infrared spectra of CaP compounds, the characteristic peaks associated with the P-O stretching vibrations appeared in the wavelength range 1200 to 900 cm^−1^, while the bands associated with the O-P-O bending vibrations appeared between 650 and 400 cm^−1^.

**Figure 4:**
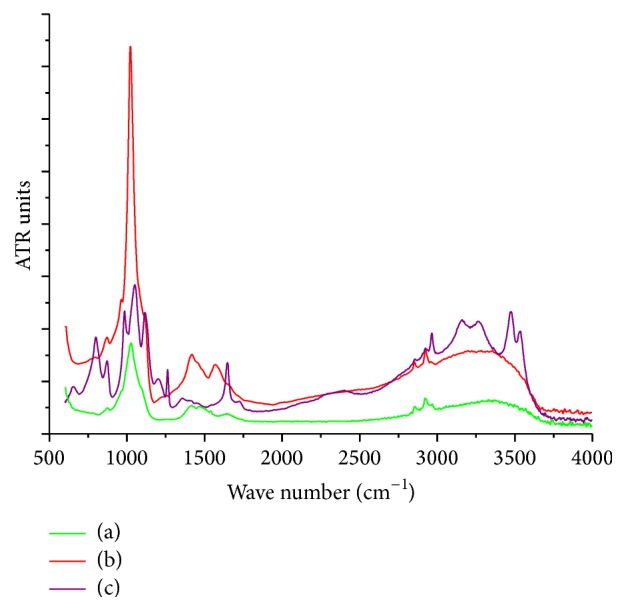
FTIR spectra of CaP coating at different electrolytic concentrations: group 10^−4^ (a), group 10^−3^ (b), and group 10^−2^ (c).

As shown in Figure 4, the FTIR spectra of CaP coatings were obtained using different concentrations of the electrolyte. The asymmetric stretching vibration peaks of PO_4_^3−^ ion appeared in the wavelength range 1010–1025 cm^−1^; these peaks were detected in the FTIR spectra of groups 10^−3^ and 10^−4^. The peak intensity of the group 10^−3^ was significantly higher than that of group 10^−4^, indicating that the group 10^−3^ had more PO_4_^3−^ ions and a thicker coating than group 10^−4^.

These findings revealed consistent conclusions regarding the XRD patterns and the SEM morphological analysis. The number of peaks detected in the group 10^−2^ was higher than that in the other groups, indicating that the coating of group 10^−2^ contained different functional groups. The characteristic peaks of group 10^−2^ were located at 1047 cm^−1^, 1117 cm^−1^, and 1198 cm^−1^; these peaks were attributed to the asymmetric stretching vibrations of the phosphate groups [31]. The absorption band that appeared in the 2964–3570 cm^−1^ region was attributed to the O-H stretching vibrations. The absorption peak near 1647 cm^−1^ was attributed to the bending vibration of crystalline water, indicating the presence of water in the coating. However, in the infrared spectra of groups 10^−3^ and 10^−4^, the absorption bands of water molecules were absent, indicating that these two compounds were anhydrous. Thus, the FTIR spectra of group 10^−2^ clearly represented the characteristic spectra of DCPD. Likewise, the absorption peaks of CO_3_^2−^ were detected at 1358 cm^−1^, 870 cm^−1^, and 650 cm^−1^.

#### 3.3.3. Hydrophilic Analysis

**Figure 5:**
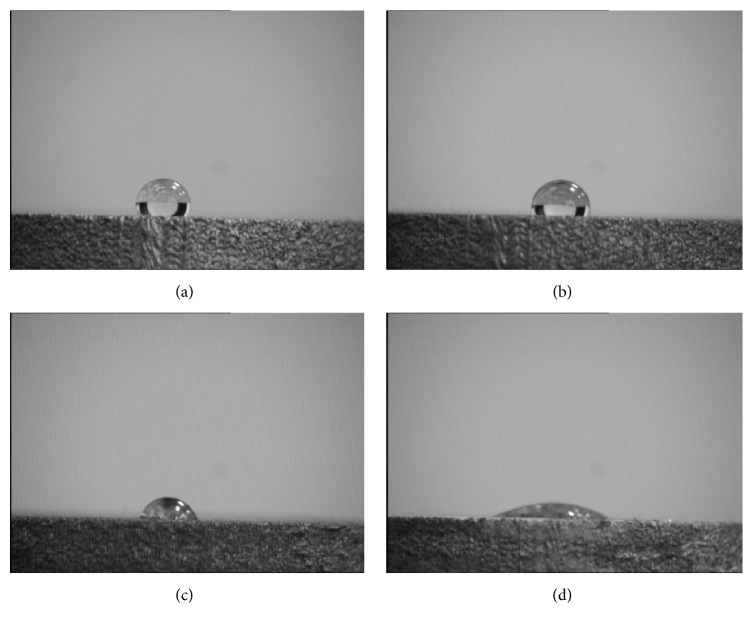
Contact angle of various study groups: control group (a), group 10^−4^ (b), group 10^−3^ (c), and group 10^−2^ (d).

The wettability of the scaffolds was examined by static water contact angles analysis (Figure 5). The contact angle in control group was 122°, which implied the Ti implant surface appeared to be hydrophobic. The surface contact angles were 104°, 76°, and 17° for the groups 10^−4^, 10^−3^, and 10^−2^, respectively. The contact angles in groups 10^−2^ and 10^−3^ were less than 90°, indicating these surfaces were hydrophilic. There were statistically significant differences among the four groups (*P* < 0.05).

### 3.4. Preliminary Evaluation of Biological Properties

**Figure 6:**
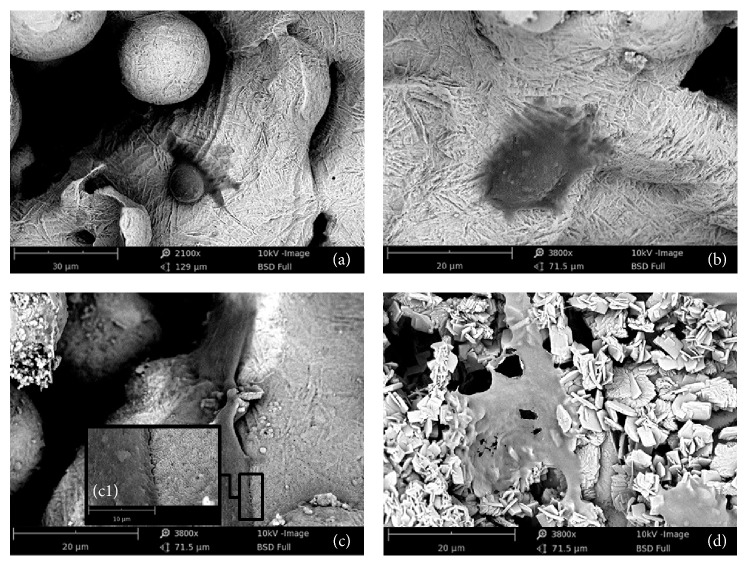
SEM micrographs of MG63 cells cultivated on the bare Ti surface (a) and on the CaP coatings at different electrolytic concentrations: group 10^−4^ (b), group 10^−3^ (c), and group 10^−2^ (d). The adherence between cell and porous surface was shown in high magnification (c1).

We determined the morphology of MG63 cell line cultured on the pure Ti and coated DLMF implants. The SEM micrographs indicated that the morphologies of cultured MG63 cells appeared to be significantly different (Figure 6). On the pure Ti implant surface, the MG63 cells presented a round cell body and a flat cytoplasm with few pseudopodia, indicating that the attachment and spreading of cells on this surface were difficult due to the hydrophobic nature. In group 10^−4^, the adhered cells stretched better on the CaP coated surface with a relatively ellipse cytoplasm. In contrast, the cells in groups 10^−3^ and 10^−2^ spread better as they had an exuberant central area and several spindle projections on the CaP coated surfaces. Under high magnification (c1), we observed abundant cytoplasmic extensions, microfilaments, and thick pseudopodia that strongly adhered to the rough and porous surface in group 10^−3^.

## 4. Discussion

The electrochemical deposition is conducted by dissolving phosphate salts and calcium salts in deionized water. In this process, the electrolyte constantly produces OH^−^ ions and H_2_ gas near the cathode. The ions released from the supersaturated solution of calcium phosphate accumulate on the surface of the specimens to produce a CaP coating [32].

Previous studies have reported that when CaP coating is produced by electrochemical deposition, the coating usually contains calcium phosphate dihydrate (DCPD), octacalcium phosphate (OCP), and HAP in higher proportions. A small amount of tricalcium phosphate (TCP) may also present in this coating. In this case, DCPD has the morphology of flat crystal wafers [33], while HAP has the morphology of thin needles. Further, electrolytic solutions containing more than 1 mM of Ca^2+^ ions are considered high concentration solutions, while those containing less than 1 mM of Ca^2+^ ions are considered low concentration solutions [34]. The concentration of ions can influence the crystallization process both dynamically and thermodynamically. According to a previous study [35], using electrolytic solutions of high concentrations, we obtained a deposition of DCPD and OCP with loose and irregular morphology. The deposition then transforms into HAP by conducting a postprocessing heat treatment. In contrast, using electrolytic solutions of low concentrations, we directly obtained a deposition of pure HAP, having uniform, compact, and controllable crystalline morphology. In this case, the XRD patterns showed that the diffraction peaks of group 10^−2^ were different from those of groups 10^−3^ and 10^−4^. Compared with the standard HAP pattern, the coating in the group 10^−2^ was predominantly DCPD. DCPD crystals are monoclinic [36], considered as the precursors of HAP [37]; they may transform into HAP by a postprocessing heat treatment. However, DCPD has a less stable structure than HAP due to its lower crystallinity [38]. Moreover, DCPD undergoes faster degradation, leading to premature loss of integrity in the coating and eventual implant failure.

Some researchers have reported that ceramic coatings bond with natural bone through a bone-like apatite layer between implant surface and the host bone [39]. FTIR is used to analyze the different functional groups existing in this coating. The bonds have been attributed to the peaks that appear in the FTIR spectra [40]. FTIR spectra showed that the absorption peaks of CO_3_^2−^ appeared around the following wavelengths: 1500, 1420, and 870 cm^−1^. Therefore, we inferred that, during the deposition, CO_3_^2−^ entered the HAP lattice and formed a carbonated hydroxyapatite, which is known as “bone-like apatite.” Scientists consider the presence of CO_3_^2−^ leads to the formation of carbonated hydroxyapatite, which has better osteoinductive ability as its composition is similar to that of natural bone.

The superficial contact angle also determines the performance of this biomaterial coating. We studied the hydrophilicity of material surfaces and found that smaller contact angles favor hydrophilicity in materials and consequently increase the cellular affinity [41]. After implantation, the surface of the implants is covered with water molecules [42]. One side of the water molecule adheres to the implant surface while the other side is absorbed by hydrated ions, such as Cl^−^, Na^+^, and Ca^2+^. This is followed by adherence to osteogenesis-related proteins and osteoblasts. The adherence strength strongly depends on the surface properties of the implant [43]. The hydrophilicity of the materials is closely related to the number of OH^−^ groups presented on the surface [44]. Meanwhile, the Ti-OH groups have a significant effect on the formation of apatite, which contributes to the deposition of CaP coating that bonds with the natural bone [45]. Accordingly, both plasma proteins and osteoblastic cells have stronger adhesion to hydrophilic surfaces in both in vitro and in vivo experiments. The great compatibility between hydration shell and water ensures that HAP has favorable hydrophilic properties [46]. Therefore, CaP coating improves the surface of a specimen by decreasing the contact angles, thus increasing the wettability and interface energy of the surface. Previous studies have reported that an increase in interface energy improves the active sites presented on the surface of titanium, thereby enhancing the cellular adhesion and extension [47].

In this research study, comparing a bare DLMF surface, MG63 cells spread and stretched better on the porous network structure of the CaP coated implants in group 10^−3^ and group 10^−2^. Accordingly, the porous structure of CaP coating enhances the surface area of Ti implant, offering more adhesive sites/motifs for the osteoblasts. The CaP ions released from the coatings provide a microenvironment with a higher affinity for culture of osteoblastic cells. Furthermore, the CaP coating provides adhesion sites for proteins and hydrophilic ions (Cl^−^ and Na^+^), thereby facilitating the adhesion and migration of cells at the surface and osteogenic activity of cultured cells at an early stage.

## 5. Conclusion

By using the electrochemical deposition, porous CaP coated DLMF implants were produced in three different concentrations of the electrolyte. XRD and FTIR analysis demonstrated the coating was predominantly constituted of HAP and well-ordered in group 10^−3^. Moreover, the hydrophilic surface and cellular affinity were also significantly improved. By analyzing and evaluating the surface properties we have provided further knowledge on the electrolytic concentration effect, which is critical for improving CaP coatings on Ti implants in the future.

## Figures and Tables

**Table 1:** Experimental groups of different electrolytic concentrations.

Electrolyte	Group 10^−2^	Group 10^−3^	Group 10^−4^	Control group
Ca(NO_3_)_2_·4H_2_O	6.0 × 10^−2^ mol	6.0 × 10^−3^ mol	6.0 × 10^−4^ mol	
(NH_4_)H_2_PO_4_	3.6 × 10^−2^ mol	3.6 × 10^−3^ mol	3.6 × 10^−4^ mol	Ti implant
NaNO_3_	—	0.1 mol	0.1 mol	
